# Effects of High-Intensity Interval Training (HIIT) on Patients with Musculoskeletal Disorders: A Systematic Review and Meta-Analysis with a Meta-Regression and Mapping Report

**DOI:** 10.3390/diagnostics12102532

**Published:** 2022-10-19

**Authors:** Ferran Cuenca-Martínez, Núria Sempere-Rubio, Clovis Varangot-Reille, Josué Fernández-Carnero, Luis Suso-Martí, Patricio Alba-Quesada, Roy La Touche

**Affiliations:** 1 Exercise Intervention for Health Research Group (EXINH-RG), Department of Physiotherapy, University of Valencia, 46022 Valencia, Spain; 2 UBIC, Department of Physiotherapy, Faculty of Physiotherapy, Universitat de València, 46010 Valencia, Spain; 3 Department of Physical Therapy, Occupational Therapy, Rehabilitation and Physical Medicine, Rey Juan Carlos University, 28933 Madrid, Spain; 4 Motion in Brains Research Group, Institute of Neuroscience and Sciences of the Movement (INCIMOV), Centro Superior de Estudios Universitarios La Salle, Universidad Autónoma de Madrid, 28049 Madrid, Spain; 5 Departamento de Fisioterapia, Centro Superior de Estudios Universitarios La Salle, Universidad Autónoma de Madrid, 28049 Madrid, Spain; 6 Instituto de Neurociencia y Dolor Craneofacial (INDCRAN), 28003 Madrid, Spain

**Keywords:** high-intensity interval training, musculoskeletal pain, pain intensity, VO_2_ max, disability, quality of life

## Abstract

The aim was to assess the impact of high-intensity interval training (HIIT) on patients with musculoskeletal disorders. We conducted a search of Medline, Embase, PEDro, and Google Scholar. We conducted a meta-analysis to determine the effectiveness of HIIT on pain intensity, maximal oxygen consumption (VO_2_ max), disability, and quality of life (QoL). We employed the GRADE and PEDro scales to rate the quality, certainty, and applicability of the evidence. Results showed significant differences in pain intensity, with a moderate clinical-effect (SMD = −0.73; 95% CI: −1.40–−0.06), and in VO_2_ max, with a moderate clinical-effect (SMD = 0.69; 95% CI: 0.42–0.97). However, the meta-analysis showed no statistically significant results for disability (SMD = −0.34; 95% CI: −0.92–0.24) and QoL (SMD = 0.40; 95% CI: −0.80–1.60). We compared HIIT against other exercise models for reducing pain intensity and increasing VO_2_ max. The meta-analysis showed no significant differences in favour of HIIT. Meta-regression analysis revealed that pain intensity scores were negatively associated with VO_2_ max (R^2^ = 82.99%, *p* = 0.003). There is low-moderate evidence that the HIIT intervention for patients with musculoskeletal disorders can reduce pain intensity and increase VO_2_ max but has no effect on disability and QoL. Results also showed that HIIT was not superior to other exercise models in reducing pain intensity and increasing VO_2_ max.

## 1. Introduction

Musculoskeletal pain is an important public health issue because of its impact on quality of life (QoL) and the disability it can represent [1]. More than 20% of the world’s population is affected by painful conditions, contributing to the high consumption of healthcare resources [2]. Pain management can be approached from several perspectives, both pharmacological and non-pharmacological, the latter of which includes physical agents, manual therapy, psychosocial interventions, patient education, and exercise training [3,4].

Exercise therapy has been reported to be highly effective in managing patients with musculoskeletal pain [5] and has been shown to produce hypoalgesia by releasing beta-endorphins or endocannabinoids [6,7,8]. Exercise therapy also interacts with the autonomic, cognitive, and affective aspects of pain [9,10]. For example, a recent meta-analysis found that aerobic exercise led to reduced pain intensity, duration, and frequency as well as improved QoL for patients with migraines [11].

The effects of high-intensity interval training (HIIT) on pain tolerance and threshold have sparked interest among the scientific community concerned with pain [12,13]. As described by Andreato, HIIT is a form of training that alternates high-intensity exercises at 90% or more of the maximal oxygen consumption (VO_2_ max) (or ≥80% VO_2_ max for the clinical population) with recovery periods, repeating the exercise several times [14]. A number of articles have recently shown that HIIT could improve pain-related clinical variables in patients with musculoskeletal disorders [15,16,17]. To date, systematic reviews on HIIT have mainly focused on patients with cardiovascular diseases, cancer, or obesity, where HIIT has shown great effectiveness in modifying cardiorespiratory variables [18,19,20]. Picavet et al. found that disability and quality of life are commonly affected in patients with musculoskeletal disorders [1]. This work prompted us to include these two variables in our study, with the objective of evaluating the role of this therapeutic exercise model on this clinical population of patients with musculoskeletal disorders. In addition to this, we wanted to include the pain intensity variable because almost 1/5 of the world’s population lives with clinical conditions that involve pain [2]. Finally, we also wanted to include the variable VO_2_ max because it is an objective variable and, in addition, it is the gold standard for assessing cardiorespiratory fitness, which seems to be affected in patients with musculoskeletal disorders with associated pain [21]. As far as we know, no published review has assessed the effects of HIIT on clinical and cardiorespiratory variables in patients with musculoskeletal disorders and pain.

Therefore, the main aim of the present study was to develop a systematic review and meta-analysis to assess the effectiveness of HIIT on pain intensity, maximal oxygen consumption, disability, and health-related QoL for patients with musculoskeletal disorders. 

## 2. Materials and Methods

This systematic review and the meta-analysis were performed according to the Preferred Reporting Items for Systematic Reviews and Meta-analysis (PRISMA) guidelines described by Moher [22]. The protocol of this systematic review and meta-analysis was registered in an international registry prior to starting the review (Prospero: CRD42020216298 (5 November 2020)).

### 2.1. Inclusion Criteria

The selection criteria used in this systematic review and meta-analysis were based on methodological and clinical factors, such as the Population, Intervention, Control, Outcomes, and Study Design (PICOS) described by Stone [23]. 

#### 2.1.1. Population

The participants selected for the studies were patients older than 18 years with any kind of musculoskeletal disorder. The participants’ gender was irrelevant.

#### 2.1.2. Intervention and Control

The intervention was the HIIT exercise modality, which could be given as an independent treatment, added to an existing intervention, or embedded in an existing intervention (e.g., usual care and treatment). For the control group, the comparators were minimal intervention, no intervention, and usual care (e.g., maintenance of the habitual daily physical activity profile, standard physical activity recommendations, physical exercise habits, and exercise intervention [excluding HIIT modality]) in combination or not with placebo interventions. In addition, we performed a sub-analysis to evaluate the effectiveness of HIIT compared with other therapeutic exercise models (e.g., moderate-intensity exercise, high-intensity continuous training, and home exercises) in those articles that, in addition to a control or comparator with no intervention or minimal intervention, presented an additional group that performed an exercise model.

#### 2.1.3. Outcomes

The measures used to assess the results and effects were pain intensity, VO_2 max_, disability, and health-related QoL.

#### 2.1.4. Study Design

We selected randomised controlled trials (RCTs), randomised parallel-design controlled trials, randomised cross-over trials, and prospective controlled clinical trials. 

### 2.2. Search Strategy

The search for studies was performed using Medline (PubMed) (1950–2020), Embase (1950–2020), PEDro (1950–2020), and Google Scholar. The first search was run on the 8 November 2020 (however, the search was updated on 31 January 2022). We used a validated search filter for retrieving studies on measurement properties in PubMed; the same filter was adapted for all other databases [24]. In addition, the search was adapted and performed in Google Scholar due to its capacity to search for relevant articles and grey literature [25,26]. No restrictions were applied to any specific language as recommended by the international criteria [27]. The search strategy combined medical subject headings (MeSH) and non-MeSH terms, adding a Boolean operator (OR and/or AND) to combine them. The terms were as follows: “Hig- Intensity Interval Training”, “High-Intensity Interval Trainings”, “Interval Training, High-Intensity”, “Interval Trainings, High Intensity”, “Training, High-Intensity Interval”, “Trainings, High-Intensity Interval”, “High-Intensity Intermittent Exercise”, “Exercise, High-Intensity Intermittent”, “Exercises, High-Intensity Intermittent”, “High-Intensity Intermittent Exercises”, “Sprint Interval Training”, “Sprint Interval Trainings”, “Pain”, “Chronic Pain”, “Musculoskeletal Pain”, “Pain intensity”, “Disability”, “Quality of Life”, “VO_2_ max”, “Maximal Oxygen Consumption”, and “Maximal Oxygen Uptake”.

Two independent reviewers (F.C.-M. and J.F.-C.) conducted the search using the same methodology, and the differences were resolved by consensus. Additionally, meticulous manual searches were performed, including journals that have published articles related to the topic of this review as well as reference lists of the included studies. The reference sections of the original studies were screened manually. To remove duplicates, we employed the citation management software Mendeley (Mendeley desktop v1.17.4, Elsevier, New York, NY, USA) and hand-checked the citations [28]. 

### 2.3. Selection Criteria and Data Extraction

First, two independent reviewers (F.C.M. and L.S.M.), who assessed the relevance of the RCTs regarding the study questions and aims, performed a data analysis, which was performed based on information from the title, abstract, and keywords of each study. If there was no consensus or the abstracts did not contain sufficient information, the full text was reviewed. In the second phase of the analysis, the full text was used to assess whether the studies met all the inclusion criteria. Differences between the two independent reviewers were resolved by a consensus process moderated by a third reviewer [29]. Data described in the results were extracted by means of a structured protocol that ensured that the most relevant information was obtained from each study [30].

### 2.4. Methodological Quality Assessment

We used the Cochrane Handbook for Systematic Reviews of Interventions version 5.1.0 to assess the risk of bias in the included studies [30]. The assessment tool covers a total of 7 domains: (1) random sequence generation (selection bias), (2) allocation concealment (selection bias), (3) blinding of participants and personnel (performance bias), (4) blinding of outcome assessments (detection bias), (5) incomplete outcome data (attrition bias), (6) selective reporting (reporting bias), and (7) other biases. Bias was assessed as low risk, high risk, or unclear risk.

The studies’ methodological quality was assessed using the PEDro scale [31], which assesses the internal and external validity of a study and consists of 11 criteria: (1) specified study eligibility criteria, (2) random allocation of patients, (3) concealed allocation, (4) measure of similarity between groups at baseline, (5) patient blinding, (6) therapist blinding, (7) assessor blinding, (8) fewer than 15% dropouts, (9) intention-to-treat analysis, 10) intergroup statistical comparisons, and 11) point measures and variability data. The methodological criteria were scored as follows: yes (1 point), no (0 points), or do not know (0 points). The PEDro score for each selected study provided an indicator of the methodological quality (9–10 = excellent; 6–8 = good; 4–5 = fair; 3–0 = poor) [32]. We used the data obtained from the PEDro scale to map the results of the quantitative analyses. 

Two independent reviewers (F.C.-M. and L.S.-M.) examined the quality of all the selected studies using the same methodology. Disagreements between the reviewers were resolved by consensus with a third reviewer. The concordance between the results (inter-rater reliability) was measured using Cohen’s kappa coefficient (κ) as follows: (1) κ > 0.7 indicated a high level of agreement between assessors; (2) κ = 0.5–0.7 indicated a moderate level of agreement; and (3) κ < 0.5 indicated a low level of agreement) [33].

### 2.5. Evidence Map 

We created a visual map of the scientific evidence for each article to visually display the information as a bubble plot. The review information is based on 3 dimensions:

1. Type of outcome measure (bubble colour): The bubble colour represents the variables (pain intensity, blue; VO_2 max_, violet; disability, green; QoL, black).
2. Variable (x-axis): We employed the calculation of effect sizes.
3. Effect (y-axis): Each of the reviews was classified according to its methodological quality using the PEDro scale.
4. Statistically significant differences: Articles with statistically significant differences were marked with white dots.

### 2.6. Certainty of Evidence 

The certainty of evidence analysis was based on classifying the results into levels of evidence according to the Grading of Recommendations, Assessment, Development, and Evaluation (GRADE) framework, which is based on five domains: study design, imprecision, indirectness, inconsistency, and publication bias [34]. The assessment of the five domains was conducted according to GRADE criteria [35,36]. Evidence was categorised into the following four levels accordingly: (a*)* High quality. Further research is very unlikely to change our confidence in the effect estimate. All five domains are also met; (b) Moderate quality. Further research is likely to have an important impact on our confidence in the effect estimate and might change the effect estimate. One of the five domains is not met; (c) Low quality. Further research is very likely to have a significant impact on our confidence in the effect estimate and is likely to change the estimate. Two of the five domains are not met; and, finally, (d) Very low quality. Any effect estimates are highly uncertain. Three of the five domains are not met [35,36].

For the study design domain, the recommendations were downgraded one level in the event there was an uncertain or high risk of bias and serious limitations in the effect estimate (more than 25% of the participants were from studies with fair or poor methodological quality, as measured by the PEDro scale). In terms of inconsistency, the recommendations were downgraded one level when the point estimates varied widely among studies, the confidence intervals showed minimal overlap, or when the I^2^ was substantial or large (greater than 50%). At indirectness domain recommendations were downgraded when severe differences in interventions, study populations or outcomes were found (the recommendations were downgraded in the absence of direct comparisons between the interventions of interest or when there are no key outcomes, and the recommendation is based only on intermediate outcomes or if more than 50% of the participants were outside the target group). For the imprecision domain, the recommendations were downgraded by one level if there were fewer than 300 participants for the continuous data [37]. 

### 2.7. Data Synthesis and Analysis

The statistical analysis was conducted using MetaXL software (version 5.3 (EpiGear International, Sunrise Beach, Queensland, Australia) [38]. To compare the outcomes reported by the studies, we calculated the standardised mean difference (SMD) over time and the corresponding 95% confidence interval (CI) for the continuous variables. The statistical significance of the pooled SMD was examined as Hedges’ g to account for a possible overestimation of the true population effect size in the small studies [39]. 

We used the same inclusion criteria for the systematic review and the meta-analysis and included three additional criteria: (1) In the results, there was detailed information regarding the comparative statistical data of the exposure factors, therapeutic interventions, and treatment responses; (2) the intervention was compared with a similar control group; and (3) data on the analysed variables were represented in at least three studies.

The estimated SMDs were interpreted as described by Hopkins et al. [40], that is, we considered that an SMD of 4.0 represented an extremely large clinical effect, 2.0–4.0 represented a very large effect, 1.2–2.0 represented a large effect, 0.6–1.2 represented a moderate effect, 0.2–0.6 represented a small effect, and 0.0–0.2 represented a trivial effect. We estimated the degree of heterogeneity among the studies using Cochran’s Q statistic test (a *p*-value < 0.05 was considered significant) and the inconsistency index (I^2^) [40]. We considered that an I^2^ > 25% represented small heterogeneity, I^2^ > 50% represented medium heterogeneity, and I^2^ > 75% represented large heterogeneity [41]. The I^2^ index is a complement to the Q test, although it has the same problems of power with a small number of studies [41]. When the Q-test was significant (*p* < 0.1) and/or the result of I^2^ was >75%, there was heterogeneity among the studies, and the random-effects model was conducted in the meta-analysis. To detect publication bias and to test the influence of each individual study, we performed a visual evaluation of the Doi plot [42], seeking asymmetry. We also performed a quantitative measure of the Luis Furuya-Kanamori (LFK) index, which has been shown to be more sensitive than the Egger test in detecting publication bias in a meta-analysis of a low number of studies [43]. An LFK index within ±1 represents no asymmetry, exceeding ±1 but within ±2 represents minor asymmetry, and exceeding ±2 involves major asymmetry. To test each study’s influence, we visually examined the forest plot and performed an exclusion sensitivity analysis. Lastly, we applied a meta-regression analysis to analyse the relationship between pain intensity and VO_2_ max variables using a random effects model employing the effect size statistic (Hedges’ *g*) of the pain intensity scores to correlate with the VO_2_ max scores [44].

## 3. Results

**Figure 1:**
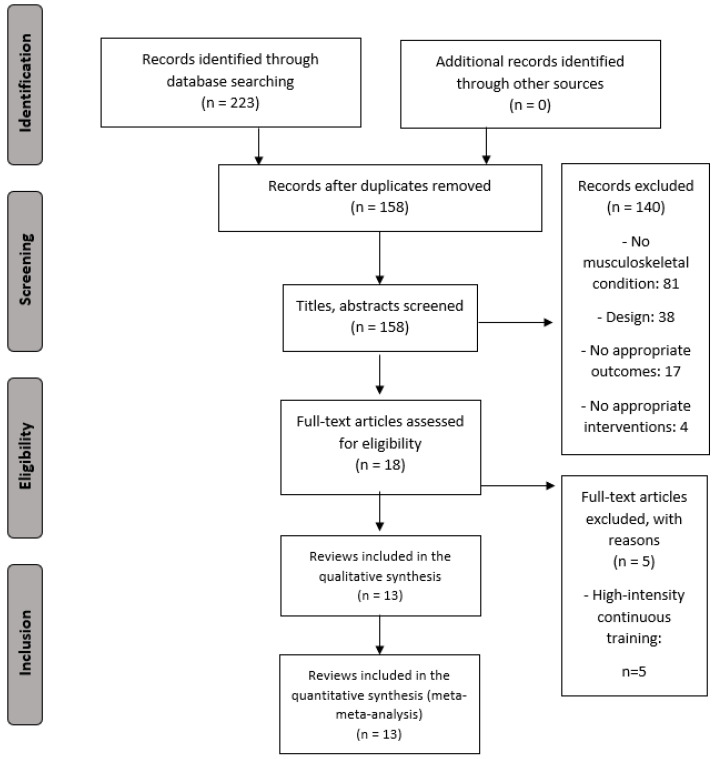
PRISMA Flowchart for selecting studies.

The study search strategy was presented in the form of a flow diagram (Figure 1).

### 3.1. Characteristics of the Included Studies

The patients were diagnosed with a persistent musculoskeletal pain condition [2 knee osteoarthritis studies [45,46], two axial spondylarthritis studies [16,47], three studies on chronic nonspecific low back pain [17,48,49], one study on episodic migraineurs [50], one study on fibromyalgia [15], one study on subacromial pain syndrome [51], one study on rheumatoid arthritis and adult-juvenile idiopathic arthritis [52], and one study on general persistent pain condition with previous trauma [53], and all of them evaluated pain intensity, VO_2_ max, disability, and health-related QoL. Table 1 lists the descriptive characteristics of the included studies.

### 3.2. Interventions

In all groups, HIIT was compared to other types of training or interventions (including controls and no interventions), with the exception of Bressel et al. [45], which studied a single HIIT and balance training group, and Sveaas et al. (2014 & 2019) [16,47], which included an HIIT and moderate-intensity continuous training (MICT) group and another no exercise group. Of the studies referred to above, three had two groups: one HIIT group and one MICT group [15,17,46]. Atan and Karavelioğlu [15] included a third standard care group. Two other studies had only one HIIT and one standard care group [48,51]. Two studies had an HIIT group and another group that maintained the activities of daily living [52] and their usual physical activity [54]. Flehr et al. [53] had one HIIT group and one yoga group, while Verbrugghe et al. [49] studied four groups with different types of HIIT. The total duration of the intervention ranged from 6 to 12 weeks, with most studies having a frequency of two to three times per week, except for Keogh et al. [46] and Atan and Karavelioğlu [15], which had frequencies of four and five times per week, respectively. Table 2 presents extensive details on the intervention characteristics of the included studies.

### 3.3. Methodological Quality Results

**Figure 2:**
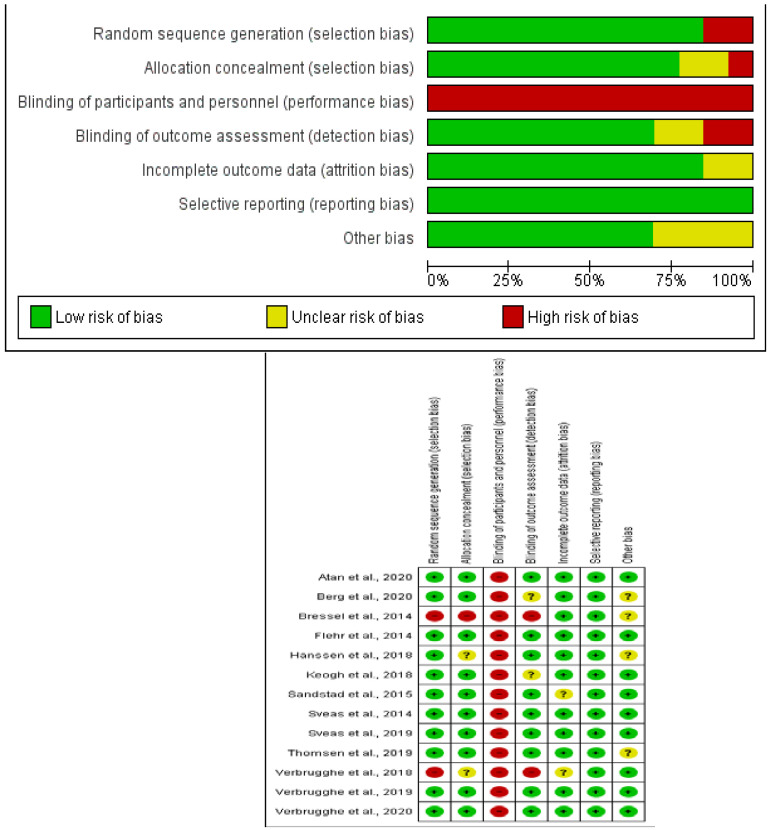
Risk of bias summary. Review authors’ judgements about each risk of bias item for each included study (Risk of Bias scale) and risk of bias graph. Review authors’ judgements about each risk of bias item presented as percentages across all included studies (Risk of Bias scale).

We evaluated the studies’ quality with the Cochrane assessment tool. Most of the studies had a low risk of selective reporting bias. The domain with the highest percentage of studies with a high risk of bias was the blinding of participants and personnel (performance bias). Figure 2 shows the risk of bias summary and risk of bias graph. The inter-rater reliability of the methodological quality assessment was high (κ = 0.787). All of the studies had an excellent or good methodological quality, except the one by Bressel et al. [45] Due to the nature of the interventions, none of the studies performed blinding of the patients or evaluators. Table 3 lists the PEDro scores for each study. The inter-rater reliability of the methodological quality assessment between assessors was high (κ = 0.815).

### 3.4. Evidence Map

**Figure 3:**
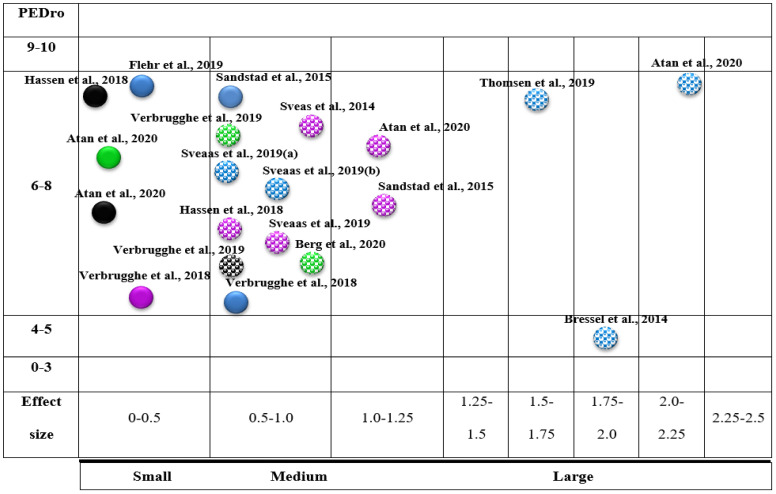
A mapping of included studies based on effect size. Blue, Pain intensity; Violet, VO_2 max_; Green, Disability; Black, Quality of Life. Bubbles marked with white dots indicate statistically significant differences (*p* < 0.05).

Figure 3 presents the results of the evidence map for the included studies.

### 3.5. Meta-Analysis Results

#### 3.5.1. Pain Intensity

**Figure 4:**
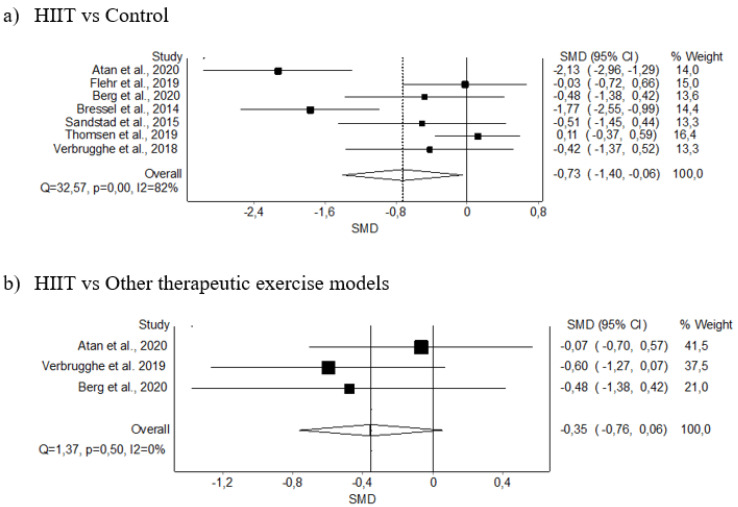
Synthesis forest plot of pain intensity variable. The forest plot summarises the results of the included studies (sample size, standardised mean differences [SMDs], and weight). The small boxes with the squares represent the point estimate of the effect size and sample size. The lines on either side of the box represent a 95% confidence interval (CI).

**Figure A1:**
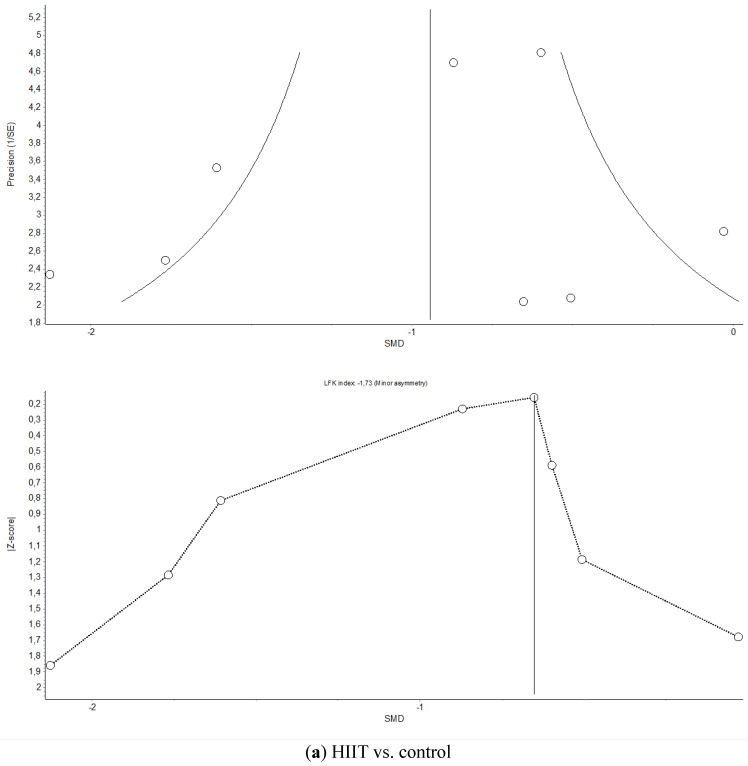

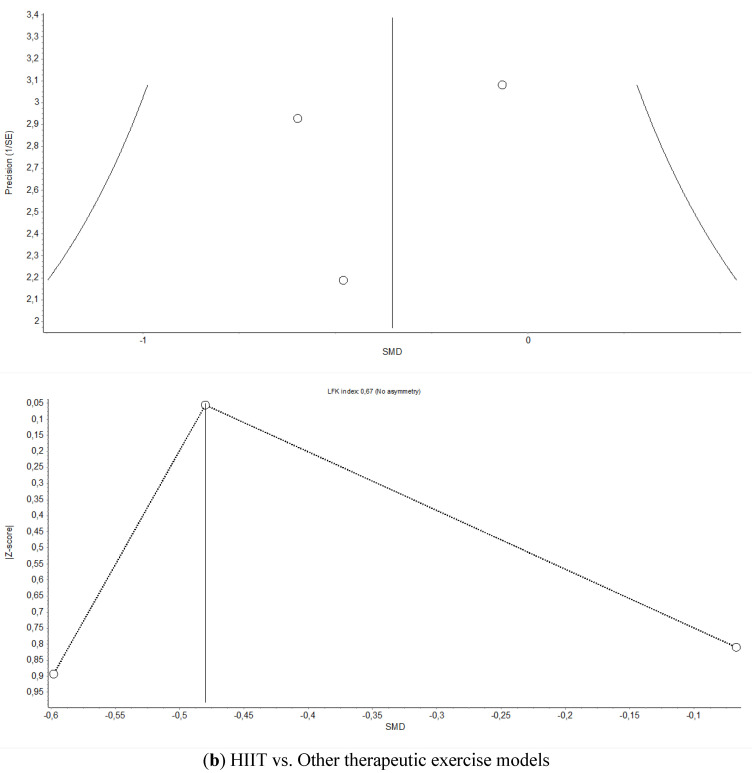
Synthesis funnel and Doi plot (LFK index) for pain intensity to assess the presence of publication bias.

The meta-analysis showed statistically significant differences for the HIIT intervention, with a moderate clinical effect in seven studies (SMD: −0.73; 95% CI −1.40–−0.06; *p* < 0.05) but with evidence of significant heterogeneity (Q = 32.57, *p* < 0.001, I^2^ = 82%). The shape of the funnel and DOI plot did not present asymmetry, and the LFK index showed minor asymmetry (LFK, −1.73) indicating a low risk of publication bias (Figure 4A and Figure A1). The certainty of the evidence was low, showing that HIIT likely decreases pain intensity, having been downgraded due to imprecision (sample size < 300) and inconsistency (I^2^ = 82%) (Table 4).

**Figure A2:**
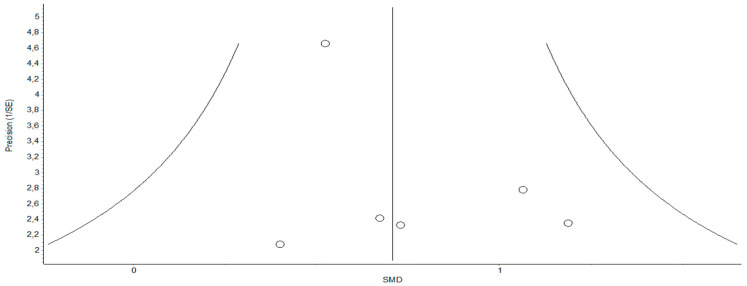

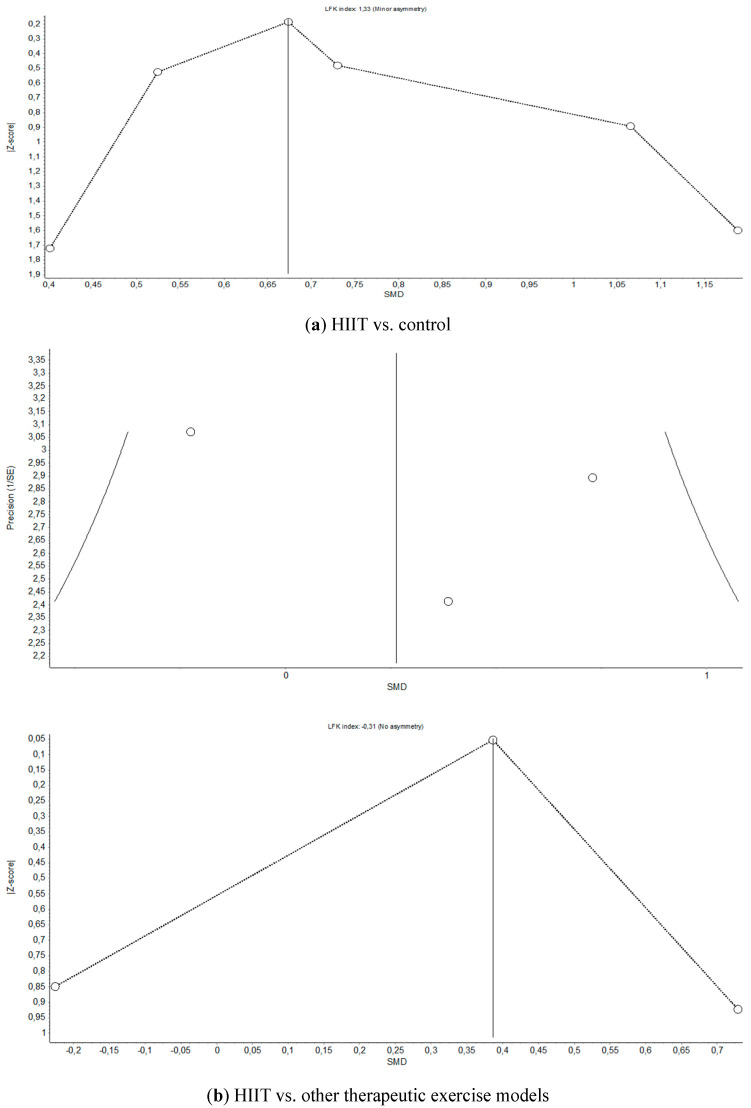
Synthesis funnel and Doi plot (LFK index) for VO_2_ max to assess the presence of publication bias.

Regarding the sub-analysis comparing HIIT against other therapeutic exercise models, the meta-analysis showed no significant differences for the HIIT intervention in 3 studies (SMD: −0.35; 95% CI −0.76–0.06, *p* ≥ 0.05) with no evidence of significant heterogeneity (Q = 1.37, *p* = 0.5, I^2^ = 0%). The shape of the funnel and DOI plot did not present asymmetry, and the LFK index showed no asymmetry (LFK, 0.67) indicating a very low risk of publication bias (Figure 4B and Figure A2).

#### 3.5.2. VO_2 max_

**Figure 5:**
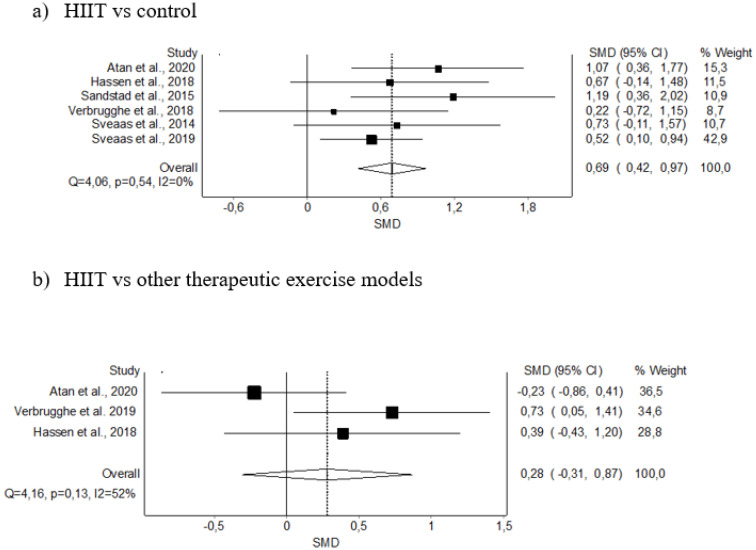
Synthesis forest plot of VO_2 max_ variable. The forest plot summarises the results of the included studies (sample size, standardised mean differences [SMDs], and weight). The small boxes with the squares represent the point estimate of the effect size and sample size. The lines on either side of the box represent a 95% confidence interval (CI).

The meta-analysis showed significant differences for the HIIT intervention, with a moderate clinical effect in six studies (SMD: 0.69; 95% CI 0.42–0.97, *p* < 0.05), with no evidence of significant heterogeneity (Q = 4.06, *p* = 0.54, I^2^ = 0%). The shape of the funnel and DOI plot did not present asymmetry, and the LFK index showed minor asymmetry (LFK, 1.33) indicating a low risk of publication bias (Figure 5A and Figure A2). The certainty of the evidence was moderate, showing that HIIT probably increases VO_2_ max, having been downgraded due to imprecision (sample size < 300) (Table 4).

Regarding the subanalysis comparing HIIT against other therapeutic exercise models, the meta-analysis showed no statistically significant differences for the HIIT intervention in three studies (SMD: 0.28; 95% CI −0.31–0.87, *p* ≥ 0.05), with no evidence of significant heterogeneity (Q = 4.16, *p* = 0.13, I^2^ = 52%). The shape of the funnel and DOI plot did not present asymmetry, and the LFK index showed no asymmetry (LFK, −0.31) indicating a very low risk of publication bias (Figure 5B and Figure A2).

#### 3.5.3. Disability

**Figure 6:**
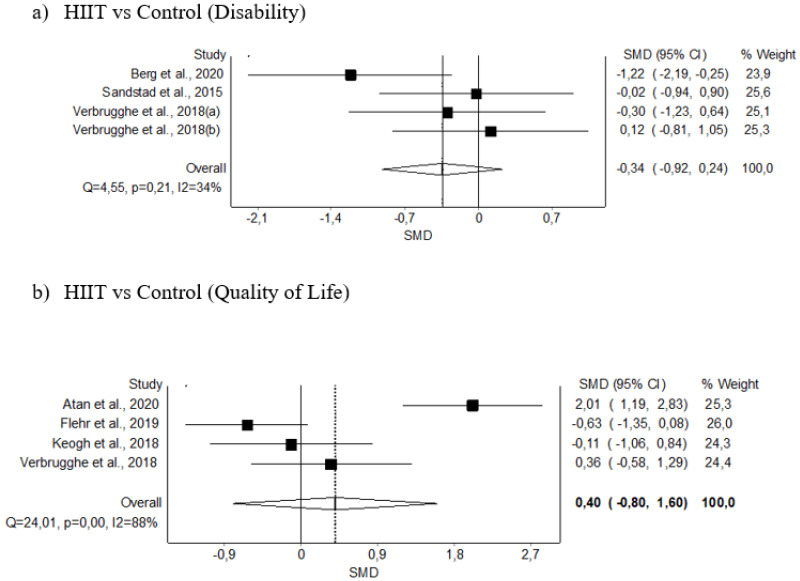
Synthesis forest plot of disability and quality-of-life variables. The forest plot summarises the results of the included studies (sample size, standardised mean differences [SMDs], and weight). The small boxes with the squares represent the point estimate of the effect size and sample size. The lines on either side of the box represent a 95% confidence interval (CI).

**Figure A3:**
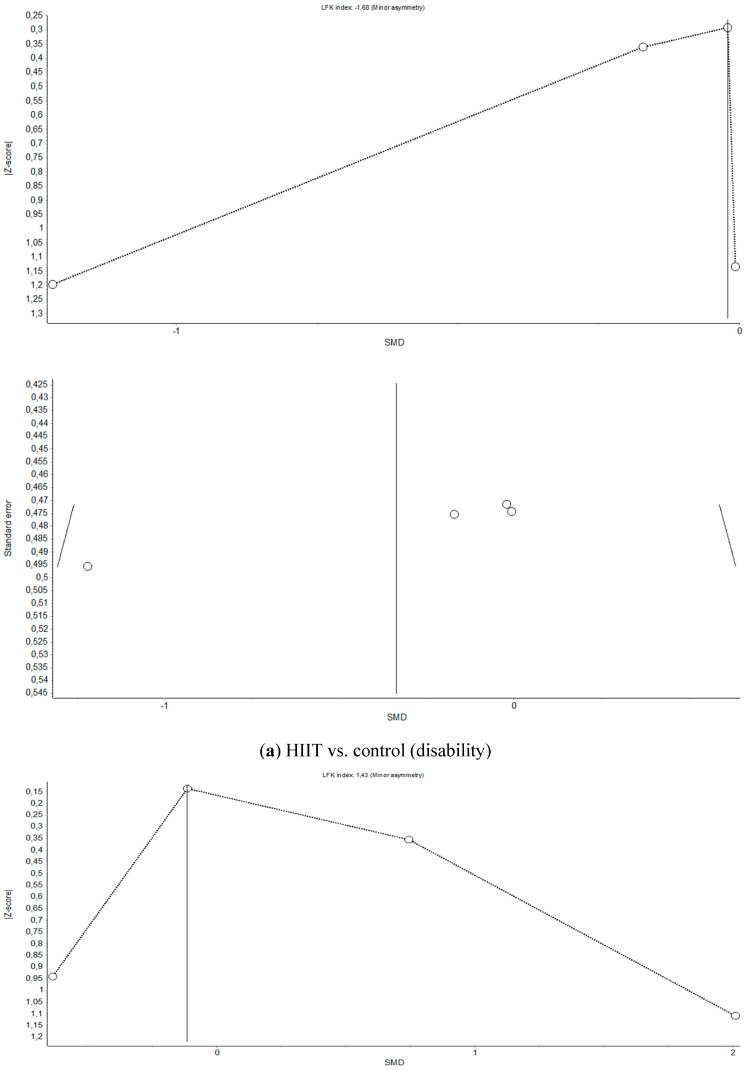

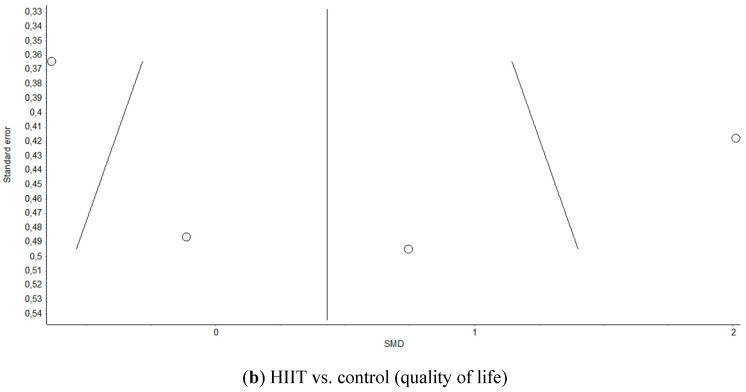
Synthesis Funnel and Doi plot (LFK index) for disability and quality-of-life variables to assess the presence of publication bias.

The meta-analysis showed no statistically significant differences for the HIIT intervention in three studies (SMD: −0.34; 95% CI −0.92–0.24, *p* ≥ 0.05), with no evidence of significant heterogeneity (Q = 4.55, *p* = 0.21, I^2^ = 34%). The shape of the funnel and DOI plot did not present asymmetry, and the LFK index showed minor asymmetry (LFK, −1.68) indicating a low risk of publication bias (Figure 6A and Figure A3). The certainty of the evidence was moderate, showing that HIIT probably does not decrease disability, being downgraded due to imprecision (sample size <300) (Table 4).

#### 3.5.4. Quality of Life

The meta-analysis showed no significant differences for the HIIT intervention in 4 studies (SMD: 0.40; 95% CI −0.80–1.60, *p* ≥ 0.05), with evidence of significant heterogeneity (Q = 24.01, *p* < 0.001, I^2^ = 88%). The shape of the funnel and DOI plot did not present asymmetry, and the LFK index showed minor asymmetry (LFK, 1.43), indicating a low risk of publication bias (Figure 6B and Figure A3). The certainty of the evidence was low, showing that HIIT likely does not increase QoL, being downgraded due to imprecision (sample size < 300) and inconsistency (I^2^ = 88%) (Table 4).

### 3.6. Meta-Regression Analysis

**Figure 7:**
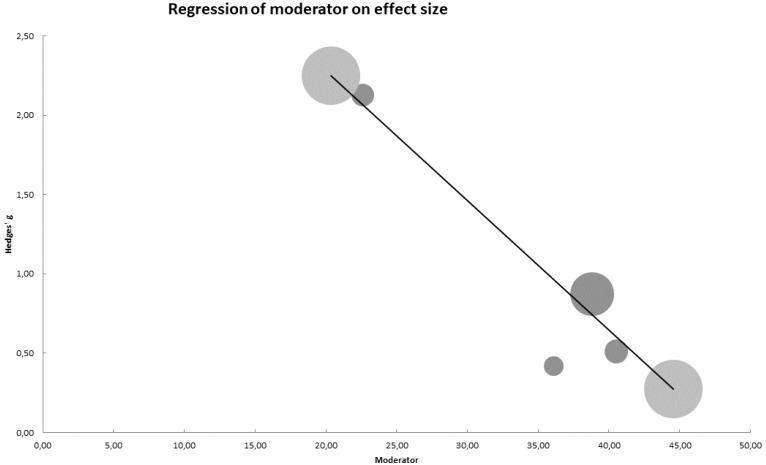
Meta-regression of pain intensity and VO_2 max_ scores. The meta-regression approach uses regression analysis to determine the influence of selected variables (the independent variables) on the effect size (the dependent variable). The large bubbles, together with the line, indicate the relationship of our model, and the small bubbles indicate their position, the relationship in the map of the effect size on the decrease in pain, on the score in the variable of maximal oxygen consumption.

In the meta-regression analysis, we explored the role of pain intensity scores in improving VO_2_ max function. The results showed that pain intensity was significantly and negatively correlated with VO_2_ max (β = −0.91; Z = −3.02; *p* = 0.003 and R^2^ = 82.99%) (Figure 7).

## 4. Discussion

Our main goal was to analyse the effect of HIIT on the VO_2 max_, pain intensity, disability, and QoL of patients with musculoskeletal disorders. Our results suggest that HIIT has a significant moderate effect size on VO_2 max_ and pain intensity but does not seem to improve the disability and QoL of patients with musculoskeletal disorders. We also found that pain intensity was negatively associated with VO_2_ max.

We found a moderate certainty of evidence of a moderate effect size of HIIT on VO_2 max_ when compared with no intervention. Several authors also found that HIIT was superior to usual care or no intervention in improving VO_2 max_ among patients with cardiovascular disorders or cancer [18,19,55]. We did not find that HIIT was superior to another exercise intervention on VO_2_ max; however, the results across systematic reviews differ [19,56,57]. It has been previously reported that HIIT induces muscular adaptations, such as mitochondrial biogenesis and increased intramuscular capillarisation [58,59] vascular adaptations, such as increased blood cell volume [60], and cardiac adaptations, such as increased cardiac output and contractility [59,61]. All of these mechanisms have been shown to play a role in VO_2 max_ [62].

We found that the patients’ pain intensity scores were negatively associated with VO_2 max_, which is an important predictor of all-cause mortality and cardiovascular disease [63,64]. It should be noted that patients with chronic pain and musculoskeletal disorders have shown an increased risk of cardiovascular and chronic disease and an increased risk of mortality due to cardiac disease [65,66]. An improvement in cardiorespiratory capacity has been shown to decrease the mortality risk by up to 16% [67,68]. HIIT appears to be an effective solution for improving patients’ cardiorespiratory capacity.

We found a low certainty of evidence of a moderate effect size of HIIT on pain intensity compared with no intervention. Geneen et al. found that physical activity appears to induce exercise-induced hypoalgesia in patients with chronic pain; however, the results were inconsistent across the various exercise modalities [69]. When compared with another exercise intervention, HIIT did not show a greater effect. It has been shown that exercise-induced hypoalgesia acts through the activation of nociceptive inhibitory pathways that release endogenous opioids and endocannabinoids [70]; however, populations with chronic pain often have exercise-induced hypoalgesia dysfunction [70,71]. Nonetheless, we found that HIIT appeared to be an effective modality for decreasing pain intensity. Patients with musculoskeletal disorders often present central sensitisation, a facilitation of the nociceptive signal in the central nervous system [72]. Quantitative sensory testing is employed to evaluate central nervous system nociceptive modulation [72]. HIIT has shown an intensity-dependent [12,13] positive effect on pain tolerance [13] and pain thresholds [12,73]. In certain conditions, the presence of an inflammatory state can increase nociceptor activity and has been associated with pain intensity [71,74,75,76]. After performing HIIT, a number of authors have found a decrease in inflammatory markers [77,78,79], such as C-reactive protein, tumour necrosis factor-alpha and interleukin-6 (IL-6), and a release of anti-inflammatory cytokines, such as IL-10 [79]. In contrast, other authors have found that HIIT induced an acute increase in IL-6 levels [80,81]; however, Pedersen proposed that this acute liberation will then induce an anti-inflammatory response [82]. Shanaki et al. observed a decrease in pro-inflammatory M1-macrophage markers and an increase in anti-inflammatory M2-macrophage markers in mice after HIIT [83]. However, not all musculoskeletal conditions show reduced pain intensity in parallel with a decrease in pro-nociceptive or inflammatory serum markers [76,84], and not all musculoskeletal conditions progress with an increased inflammatory state [76].

We found a low level of evidence of no significant effect of HIIT on QoL compared with no intervention or usual care. Mugele et al. systematically reviewed the effect of HIIT on QoL, compared with usual care, and found unclear results [19]. QoL appears to be more closely related to interpretation and catastrophising than pain intensity [85], which might explain why we observed a decrease in pain intensity with no improvement in QoL. Monticone et al. found that a multidisciplinary treatment involving cognitive-behavioural therapy and exercise results in a significant improvement in QoL, while exercise alone resulted in little change [86]. We also found moderate certainty evidence of no significant effect of HIIT on disability compared with no intervention or usual care. Kamper et al. found that a treatment involving a physical and a psychological or social component had a greater effect on disability than physical therapy alone for patients with chronic low back pain. HIIT alone might be insufficient for improving disability or QoL in musculoskeletal disorders [87].

Time constraints and pain are two of the main barriers to physical activity for patients with musculoskeletal disorders [88,89,90]. Despite similar effects on VO_2 max_ and pain intensity with other exercise types, HIIT requires less training volume to achieve similar effects in the included studies that provide the control group’s training duration [15,50]. Wewege et al. found that the most common adverse effects in patients with cardiovascular disease were musculoskeletal complaints; however, we observed that HIIT presented similar or almost no additional major or minor adverse events or pain flare-ups than no intervention or other exercise modalities [91]. Major cardiac adverse events during HIIT appear at a rate of 1 per 11,333 HIIT h in patients with cardiovascular disease [91] but with no significant difference in the overall adverse events rate between HIIT and MICT [91]. As recommended by Weston et al. if health professionals want to implement HIIT, they should evaluate patients on a case-by-case basis depending on their cardiac history [20]. Heisz et al. found that participants rated HIIT more enjoyable than MICT and that enjoyment increased with repeated HIIT when it remained constant with repeated MICT [92]. Health professionals should include HIIT in the management of musculoskeletal disorders, given that HIIT is a time-efficient, enjoyable, effective, and safe form of exercise. Finally, it is relevant to stress that it is important to prescribe exercise specifically for each patient and for each clinical condition, although in this work it has been grouped by variables, rather than by populations.

### Limitations

We found low-to-moderate quality evidence for our results. Further studies are needed on the effects of HIIT on musculoskeletal disorders to confirm our results. The sample sizes of the included studies were often very small. Future studies should include larger sample sizes to improve the quality of the evidence. Due to the lack of sufficient data and the heterogeneity among the interventions (e.g., frequency, intervention duration), we could not establish the specific effect on each musculoskeletal disorder and the optimal HIIT parameters. Due to the small number of trials, we pooled the aerobic and anaerobic HIIT training studies; future systematic reviews should evaluate them separately. Only a few studies compared the effect of HIIT against high-intensity continuous training or other types of exercise; future studies should include this type of high-intensity training.

As recommended by the American Thoracic Society/American College of Chest Physicians Statement on Cardiopulmonary Exercise Testing, we included VO_2_ peak and VO_2 max_ and used them interchangeably [93]. Quantitative sensory testing (e.g., pain pressure or thermal threshold, conditioned pain modulation, and temporal summation) is essential in pain research; future studies evaluating the effects of HIIT on musculoskeletal disorders should include these variables. In addition, no further meta-regression analysis could be performed due to the small number of articles sharing the outcomes of interest. Lastly, it is important to stress that there were 3 studies where HIIT was embedded in other exercise interventions such as balance exercise and continuous exercise. This is a clear limitation that should be considered when extrapolating the results [16,45,47].

## 5. Conclusions

There is low to moderate quality evidence that the HIIT intervention for patients with musculoskeletal disorders can improve pain intensity and VO_2 max_ but not disability and QoL. The results of the subanalyses showed that HIIT was not superior to other exercise models in improving pain intensity and VO_2 max_. Clinically, this tells us that we can implement high-intensity interval exercise models if our goal is to improve pain intensity or increase cardiorespiratory fitness through maximal oxygen consumption. However, it is important to keep in mind two aspects: changes in pain intensity may not be accompanied by improvements in the subjective perception of quality of life or disability, at least, based on the data we currently have, and second, that this exercise model was not superior to other exercise models with respect to eliciting these clinical changes. This should be considered clinically. Low sample sizes and lack of prescription parameters emphasise the need for further research on HIIT in musculoskeletal disorders for its implementation in a clinical context.

## Figures and Tables

**Table 1:** Characteristics of the included studies.

Author, Year Country	Population Disease (*n*) Age (Years) Sex (%) Diagnostic Criteria Disease Duration (Years)	Study Design—Duration Intervention(s) and Control Group (*n*)	Outcome Measured (Instrument)	Results
Atan et al., 2020 [15] Turkey	Fibromyalgia (*n* = 55) *Age*, 48.7 ± 9.1 y 100% F American College of Rheumatology 2016 diagnostic criteria *Duration,* 2.5 ± 1.6 y	Pilot ROT—6 weeks *Intervention* - HIIT (*n* = 19) - MICT (*n* = 19) *Control * Usual care (*n* = 17)	- Pain Intensity (VAS) - HRQoL (SF-36 PF, PRL, Pain, GH, V, SF, ER, MH, EWB, E/F, HC) - VO_2 max_ (mL/kg/min)	HIIT showed significant differences compared with a control group on pain intensity, VO_2 max,_ and SF-36 PF, PRL, ER, E/F, EWB, GH, and HC (*p* < 0.05) but no significant difference compared with MCT.
Berg et al., 2020 [50] Norway	Chronic SAPS (*n* = 21) *Age*, 48.1 ± 12.5 y 48% F/52% M Clinical criteria *Duration*, 3.5 ± 4.8 y	RCT—8 weeks *Intervention* HIIT + Home-exercise (*n* = 13) *Control * Home-exercise (*n* = 8)	- Pain intensity (NPA) - Disability (SPADI)	HIIT showed significant intragroup (*p* < 0.05) and intergroup differences (*p* < 0.05) compared with a control group in terms of disability but no significant difference in pain intensity.
Bressel et al., 2014 [44] United States	Knee OA (*n* = 18) *Age,* 64.5 ± 10.2 y 89% F/11% M Clinical and radiological criteria *Duration,* 6.8 ± 7.4 y	Pre-post study—6 weeks *Intervention* - HIIT + Balance training (*n* = 18) *Control* No intervention (*n* = 18)	Pain Intensity (VAS)	HIIT showed a significant improvement in pain intensity (*p* < 0.05).
Flehr et al., 2019 [52] Australia	Persistent pain condition (*n* = 32) *Age,* 30.2 ± 8 y 100% F N/R *Duration,* More than 12 months	RCT—8 weeks *Intervention* HIIT (*n* = 15) *Control* Bikram Yoga (*n* = 17)	- Pain Intensity (BPI) - HRQoL (SF-36 PF, PRL, Pain, GH, V, SF, ER, MH)	No significant difference between HIIT and Bikram Yoga in pain intensity. There was a significant intergroup difference on quality of life (SF-36 PF: *p* = 0.019; SF-36 MH: *p* = 0.005), with yoga showing higher improvement (SF-36 PF: M = 80.91; SF-36 MH: M= 63.94).
Hanssen et al., 2018 [49] Switzerland	Episodic migraine without aura (*n*= 36) *Age*, 36.8 ± 10.3 y 81% F/19% M International classification of headache disorders, 3rd ed. *Duration*, N/R	RCT—12 weeks *Intervention* - HIIT (*n* = 13) - MICT (*n* = 11) *Control Group* No intervention (*n* = 12)	VO_2 max_ (mL/kg/min)	No group × time interaction between the three groups (*p* = 0.14).
Keogh et al., 2018 [45] Australia	Knee OA (*n* = 17) *Age,* 62.4 ± 8.3 y 76% F/24% M Diagnosis by an orthopaedic surgeon *Duration*, 4.7 ± 4.6 y	Pilot RCT—8 weeks *Intervention* HIIT (*n* = 9) *Control* MICT (*n* = 8)	- Disability (WOMAC, Lequesne Index)	Both interventions demonstrated significant benefits on the WOMAC (HIIT: *p* = 0.05; MICT: *p* = 0.006) but without intergroup differences. No patient had significant improvement in the Lequesne index.
Sandstad et al., 2015 [51] Norway	RA and JIA (*n* = 27) *Age,* 33.0 ± 8.1 y 100% F Diagnosis by a rheumatologist *Duration,* N/R	Cross-over trial—10 weeks *Intervention* HIIT (*n* = 12) *Control * No intervention (*n* = 15)	- Pain Intensity (VAS) - Disability (MHAQ) - VO_2 max_ (mL/kg/min)	HIIT had a significant improvement in VO_2 max_ (*p* < 0.001) but no difference in pain intensity and disability.
Sveaas et al., 2014 [49] Norway	axSpA (*n* = 24) *Age,* 48.5 ± 12.0 y 50% F/50% M Spondyloarthritis International Society criteria *Duration,* 24.9 ± 15.8 y	Pilot RCT—12 weeks *Intervention* HIIT (*n* = 10) *Control * Usual care (*n* = 14)	VO_2 max_ (mL/kg/min)	HIIT had a significantly higher VO_2 max_ at 12 weeks than the control group (*p* < 0.001)
Sveaas et al., 2019 [16] Norway	axSpA (*n* = 97) *Age*, 46.2 ± N/R y 53% F/47% M Spondyloarthritis International Society criteria Duration, N/R	RCT—12 weeks *Intervention* HIIT (*n* = 48) *Control* No intervention (*n* = 49)	- Pain intensity (BASDAI neck/back/hip and peripheral pain) - VO_2 max_ (mL/kg/min)	HIIT significantly improves the neck/back/hips, and peripheral pain intensity, and the VO_2 max_ more than the control group (*p* < 0.001; *p* = 0.016; *p* < 0.001).
Thomsen et al., 2019 [53] Norway	PsA (*n* = 67) *Age,* 48.0 ± 11.5 y 64% F/36% M Classification of psoriatic arthritis Study group criteria *Duration,* N/R	RCT—11 weeks *Intervention* HIIT (*n* = 32) *Control * No intervention (*n* = 35)	- Pain Intensity (VAS)	HIIT showed no clear effect on pain intensity at the end of the intervention and at 9 months of follow-up.
Verbrugghe et al., 2018 [47] Belgium	Nonspecific Chronic LBP (*n* = 20) *Age*, N/R 55% F/45% M Clinical criteria *Duration,* N/R	CCT—6 weeks *Intervention* HIIT (*n* = 10) *Control* Usual care (*n* = 10)	- Pain Intensity (NPRS) - Disability (RMDQ) - HRQoL (SF-36 PF, PRL, ER, E/F, EWB, SF, Pain, GH) - VO_2 max_ (mL/kg/min)	Both groups had a reduction in disability (*p* < 0.05) with no intergroup difference. HIIT improved significantly HRQoL (SF-36 PRL, ER, SF, and Pain) (*p* < 0.05) but with no intergroup differences.
Verbrugghe et al., 2019 [17] Belgium	Nonspecific Chronic LBP (*n* = 36) *Age,* 44.2 ± 9.8 y 68% F/32% M Clinical criteria *Duration,* 11.1 ± 7.7 y	RCT—12 weeks *Intervention* HIIT (*n* = 18) *Control* MIT (*n* = 18)	- Pain Intensity (NPRS) - Disability (MODI) - VO_2 max_ (mL/kg/min)	HIIT significantly improved disability and VO_2 max_ more than MIT (*p* < 0.05). HIIT significantly reduced pain intensity (*p* < 0.05) but with no significant differences with MIT.
Verbrugghe et al., 2020 [48] Belgium	Nonspecific chronic LBP (*n* = 80) *Age,* 44.1 ± 9.7 y 58% F/42% M Clinical criteria *Duration,* 13.4 ± 9.1 y	RCT—12 weeks *Intervention* - HITCOM (*n* = 19) - HITSTRE (*n* = 21) - HITSTAB (*n* = 20) - HITMOB (*n* = 20)	- Pain Intensity (NPRS) - Disability (MODI) - VO_2 max_ (mL/kg/min)	All four HIIT groups significantly reduced pain intensity and disability and increased VO_2 max_ (*p* < 0.05), with no intergroup differences.

axSpA, axial spondyloarthritis; BPI, Brief Pain Inventory; CCT, Controlled clinical trial; E/F, energy/fatigue; ER, emotional role limitation; EWB, emotional well-being; GH, general health; HC, health change; HIIT, high-intensity interval training; HITCOM, high-intensity general resistance training, and high-intensity core strength training; HITMOB, trunk mobility exercises; HITSTAB, high-intensity core strength training; HITSTRE, high-intensity general resistance training; HRQoL, health-related quality of life; JIA, juvenile idiopathic arthritis; LBP, low back pain; MCT, moderate continuous training; MH, mental health; MHAQ, Modified Health Assessment Questionnaire; MICT, moderate-intensity continuous training; MIT, moderate-intensity training; MODI, Modified Oswestry Index; MPQ, McGill Pain Questionnaire; N/R, not reported; NPRS, Numeric Pain Rating Scale; OA, osteoarthritis; ODI, Oswestry Disability Index; PF, physical functioning; PRL, physical role limitation; PsA, psoriatic arthritis; RA, rheumatoid arthritis; RCT, randomised control trial; RMDQ, Roland-Morris Disability Questionnaire; SF-36, Short Form-36 Health Survey; SAPS, subacromial pain syndrome; SF, social functioning; SPADI, Shoulder Pain and Disability Index; V, vitality; VAS, visual analogue scale; WOMAC, Western Ontario and McMaster Universities Osteoarthritis Index.

**Table 2:** Prescription parameters extracted from each included study.

Trial	Group	Exercise Protocol (Distribution and Exercise Type)	Intensity (Pain Control during Training)	Frequency and Duration	Exercise Testing
Atan et al., 2020 [15]	HIIT (AerT) + StrT + Stretching	*Total exercise duration:* 35 min *Warmup and cooldown:* 5 min stationary cycling. *HIIT protocol:* 4 × 4 min of high-intensity stationary cycling interval alternating with 3 min cycling recovery periods. *Work/rest ratio*: [1:0.75] Followed by 10 min full body (shoulder, arm, leg, and hip) StrT, using 1–3-kg weights (1 × 8–10 rep) and 5 min stretching (4–5 × 20–30 s for each muscle group).	*Measurement:* HR_max_ (Monitorisation: N/R) *Warmup and cooldown:* 50% HR_max_ *HIIT:* *Interval:* 80–95% HR_max_ *Active Rest:* 70% HR_max_ *StrT:* N/R *Pain:* N/R	5×/week 6 weeks	Maximal cardiopulmonary test on a cycloergometer at baseline and follow-up. HR_max_, VO_2_ max, BP, workload, MET and duration-of-test were recorded.
	MICT (AerT) + StrT + Stretching	*Total exercise duration:* 55 min. *Warmup and cooldown:* 5 min stationary cycling. *MICT protocol:* 45 min continuous stationary cycling Followed by 10 min full body (shoulder, arm, leg, and hip) StrT, using 1–3-kg weights (1 × 8–10 rep) and 5 min stretching (4–5 × 20–30 s for each muscle group).	*Measurement:* HR_max_ (Monitorisation: N/R) *Warmup and cooldown:* 50% HR_max_ *MICT:* 65–70% HR_max_ *StrT:* N/R *Pain:* N/R		
	Usual Care	Recommendations regarding exercise for fibromyalgia.	N/A		
Berg et al., 2020 [50]	HIIT (StrT) + Usual Care	*HIIT protocol:* 4 × 4 min shoulder abduction-adduction at 2 Hz intervals alternating with 3 min walking rest periods *Work/Rest Ratio*: [1:0.75] If the patient was able to continue the final interval for one additional minute, the workload was increased by 250 g in the following session. *Home-based exercises:* Scapular stabilising, rotator cuff, and pain-free ROM exercises.	*Measurement:* WR_max_ *Interval:* 80% WR_max_ *Rest:* N/R *Pain:* When pain exceeds 5/10, session was ended.	3×/week 8 weeks	Time to exhaustion test during shoulder abduction-adduction. WR_max_ was recorded.
	Usual Care	*Home-based exercises:* Scapular stabilising, rotator cuff, and pain-free ROM exercises.	N/R		
Bressel et al., 2014 [44]	BalanceT + HIIT (AerT)	*Balance training:* Perturbations with water jets. Followed by: *HIIT protocol:* (Progressive increase from 1^st^ to 6^th^ week) 3 to 6 × 0.5 to 2.5 min walking (1.3 to 2.1 m/s) on an underwater treadmill interval alternating with 1 to 2.5 min walking (1.3 to 1.8 m/s) rest periods. (depth: xiphoid process) *Work/rest ratio*: [1:2; 1:1.3; 1:1; 1:1; 1:1; 1:1]	*Measurement:* RPE (Borg Scale/20) *BalanceT:* Progressive increase (from 1^st^ to 6^th^ week) from 11 to 18/20. *HIIT:* *Interval:* Progressive increase (from 1^st^ to 6^th^ week) from 13 to 19/20. *Rest*: 10/20. *Pain:* N/R	3×/week 6 weeks	N/A
Flehr et al., 2019 [52]	HIIT (StrT + AerT)	45 min functional training incorporating running, throwing, standing from a seated position, placing items overhead, and picking items up. *Warmup and demonstration:* 15 min. *Movement learning*: 15 min *HIIT protocol:* 15 min reproduction of the movement at high intensity. Four formats possible: As fast as possible, 8-exercises Tabata intervallic training followed by AerT, Maximum reps or load in a set time, or as many rounds as possible in 12 min followed by AerT	N/R *Interval:* N/R *Rest:* N/R *Pain*: N/R	3×/week 8 weeks	N/R
	Yoga	90 min Bikram Yoga class (Room at 40 °C and 40% humidity): Deep breathing, 45 to 50 min standing, stretching, and relaxation postures.	Light to moderate (according to ACSM) and sometimes vigorous. *Pain:* N/R		
Hanssen et al., 2018 [49]	HIIT (AerT)	*Warmup:* 400 m of light running on a treadmill and 2 skipping exercises *HIIT protocol:* 4 × 4 min high-intensity running on a treadmill, interval alternating with 3 min running recovery periods. *Work/rest ratio:* [1:0.75] *Cooldown:* 400 m of light running and stretching	*Measurement:* HR_max_ (HR checked using HR monitor) *Interval:* 90% to 95% HRmax (±5 bpm) *Rest:* 70% of HRmax *Pain:* N/R	2×/week 12 weeks	Maximal Cardiopulmonary test on a treadmill. Anaerobic lactate-threshold, HR_max_, RPE, and VO_2_ max were recorded.
	MICT (AerT)	*Warmup:* 400 m of light running on a treadmill and 2 skipping exercises *MICT protocol:* 45 min continuous running on a treadmill. *Cooldown:* 400 m of light running and stretching	*Measurement:* HR_max_ (HR checked using HR monitor) *MICT:* 70% HRmax (± 5 bpm) *Pain:* N/R		
	Maintain their habitual daily physical activity	N/A	N/A		
Keogh et al., 2018 [45]	HIIT (AerT)	*Warmup:* 7 min stationary cycling, with progressively increasing intensity *HIIT protocol:* 5 × 45 s high-cadence stationary cycling interval alternating with 90 s low-intensity recovery cycling. Work/Rest Ratio: [1:2] *Cooldown:* 6–7 min of light to moderate cycling.	*HIIT:* *Interval:* 110 rpm with a resistance similar or slightly higher than the rest. Intensity was defined as “an intensity at which you felt it was quite difficult to complete sentences during the exercise”. *Rest:* ∼70 rpm To avoid pain, progressive increase in initial sessions.	4×/week 8 weeks	N/R
	MICT (AerT)	*Warmup and cooldown:* Light intensity cycling for 3 min and 2 min, respectively. *MICT protocol:* 20 min continuous cycling.	*MICT:* 60–80 rpm. Intensity was defined as “An intensity at which you are able to speak in complete sentences during the exercise”. To avoid pain, progressive increase in initial sessions		
Sandstad et al., 2015 [51]	HIIT (AerT)	*Warmup:* 10 min stationary cycling at moderate intensity *HIIT protocol:* 4 × 4 min high-intensity stationary cycling interval alternating with 3 min cycling recovery periods. The speed and workload were adjusted continuously.	*Measurement:* HR_max_ (HR checked using HR monitor) *Warmup:* ~70% *Interval:* 85–95% of HRmax *Rest:* ~70% of HRmax *Pain:* N/R	2×/week 10 weeks	Maximal cardiopulmonary test on a bike. VO_2_ max and HR_max_ (defined as the highest HR during the test plus 5 bpm).
	Maintain daily life activities	N/A	N/A		
Sveaas et al., 2014 and 2019 [16,49]	HIIT (AerT) + StrT + MICT (AerT)	Twice a week, supervised HIIT and StrT: *- HIIT protocol:* 4 × 4 min walking/running on a treadmill interval alternating with 3 min of active resting. *- StrT protocol*: 20 min with external load (2–3 × 8–10 rep): Bench press or chest press machine, weighted squat or leg press machine, rowing with weights, triceps and biceps machine, and abdominal bridge. Once a week, individual interval training or MICT: 40 min of either interval training or MICT.	*Measurement:* HR_max_ (HR checked using HR monitor) *HIIT:* *Interval:* 90–95% HR_max_ Rest: 70% HR_max_ *MICT intensity:* >70% HR_max_ *Pain*: Exercises were adapted if pain was ≥ 5/10	3×/week 12 weeks	Cardiopulmonary test on a walking treadmill (modified Balke protocol). VO_2_ max and HR_max_ were recorded.
	Asked to not start exercise	N/A	N/A		
Thomsen et al., 2019 [53]	HIIT (AerT)	*Warmup:* 10 min. *HIIT protocol:* 4 × 4 min high-intensity stationary cycling interval alternating with a 3 min cycling recovery period. *Work/rest ratio:* [1:0.75] Supervised twice a week and individually once a week. Participants were instructed in using the HIIT concept by, for example, running, bicycling, or walking uphill.	*Measurement:* HR_max_ (HR checked using HR monitor) *Interval:* 85–95% HR_max_ *Rest:* 70% HR_max_ *Pain:* N/R	3×/week 11 weeks	Maximal cardiopulmonary test on a bike. VO_2_ max and HR_max_ (defined as the highest HR during the test more 5 bpm).
	Maintain daily physical activity	N/A	N/A		
Verbrugghe et al., 2018 [47]	HIIT (AerT) + High Intensity StrT	*HIIT protocol:* *-Warmup:* 5 min *-Followed by HIIT training:* 5 × 1 min high-intensity stationary cycling interval alternating with 1 min of rest. Weekly increase of interval duration by 10 s until week 6. *Work/rest ratio*: [1:1; 1.2:1; 1.3:1; 1.5:1; 1.7:1; 1.8:1] *High load whole body StrT training protocol: * 3 upper body (pulley biceps curl, pulley chest press, and pulley vertical traction behind the neck) and 3 lower body exercises (leg press, leg extension, and leg curl) with external load: 1 to 2 × 8–12 rep.	*Measurement:* VO_2_ max and 1RM (Monitorisation: N/R) *Interval:* VO_2_ max workload *Rest:* N/R *StrT:* 80% 1RM *Pain:* N/R	2×/week 6 weeks	Maximal cardiopulmonary testing (Graded exercise test) on a bike. VO_2_ max, expiratory volume, respiratory exchange ratio, and HR were recorded A 1RM test was performed for every exercise.
	Usual Physiotherapy Care	*MICT protocol:* 50 min continuous cycling, cross-training, and/or treadmill walking. *Control motor exercise:* Addressing lumbopelvic motor control impairments. *Trunk StrT:* Unstable posture corrections, plank, and bridge variations	*Measurement:* HR_max_ (Monitorisation: N/R) *MICT:* 60–65% HR_max_ *Pain:* N/R		
Verbrugghe et al., 2019 [17]	HIIT (AerT) + High-intensity Global and Core StrT	*HIIT protocol:* *-Warmup:* 5 min cycling *-HIIT Training:* 5 × 1 min high-intensity cycling interval alternating with a 1 min cycling recovery period. Weekly increase of interval duration of 10 s until week 6. *Work/rest ratio*: [1:1; 1.2:1; 1.3:1; 1.5:1; 1.7:1; 1.8:1] *High-intensity StrT:* 3 upper body (vertical traction, chest press, arm curl) and 3 lower body exercises (leg curl, leg press, leg extension) executed with external load on machines: 1 × maximum 12 rep *Core muscle training:* 6 static core exercises [glute bridge, resistance band glute clam, lying diagonal back extension, adapted knee plank, adapted knee side plank, elastic band shoulder retraction with hip hinge): 1 × 10 rep of a 10 s static hold.	*Measurement:* % VO_2_ max, %1RM and %MVC (Monitorisation: N/R) *HIIT:* *Interval:* 110 rpm at 100% VO_2_ max workload *Rest:* 75 rpm at 50% VO_2_ max workload *StrT:* 80% 1RM 5% workload increase when the participant was able to perform more than 10 reps on 2 consecutive sessions. *Core:* Between 17% and 100% MVC of m. transversus abdominis, m. multifidus, m. gluteus. Progressive increase of time and load (body weight bearing, elastic or weights). *Pain:* N/R	2×/week 12 weeks	Maximal cardiopulmonary test on a bicycle. VO_2_ max, Maximal workload, LA, and HR were recorded. Workload was updated, with a complementary cardiopulmonary test, for the last 6 weeks. 1RM testing was performed for every exercise.
	MICT (AerT) + Moderate intensity Global and Core STrT	*MICT protocol:* Cycling on a cycle ergometer. *- Warmup:* 5 min. *- MICT:* Continuous 14 min cycling at moderate intensity. Duration increased by 100 s every 2 sessions up to 22 min 40 s. *Moderate intensity Global StrT:* Same exercises as above, but at moderate intensity: 1 × 15 rep. *Moderate intensity core training:* Same exercises as above but at moderate intensity: 1 × 10 repetitions of a 10 s static hold.	*Measurement:* % VO_2_ max, %1RM and %MVC (Monitorisation: N/R) *MICT:* 90 rpm at 60% VO_2_ max workload *StrT:* 60% of 1RM *Core training:* N/R *Pain:* N/R		
Verbrugghe et al., 2020 [48]	HIIT (AerT) + Global StrT	*HIIT protocol:* *- Warmup:* 5 min cycling *- HIIT Training:* 5 × 1 min high-intensity cycling interval alternating with a 1 min cycling recovery period. Weekly increase of interval duration, of 10 s, until week 6. *Work/rest ratio*: [1:1; 1.2:1; 1.3:1; 1.5:1; 1.7:1; 1.8:1] *High-intensity StrT:* 3 upper body (vertical traction, chest press, arm curl) and 3 lower body exercises (leg curl, leg press, leg extension) executed with external load on machines: 2 × maximum 12 rep	*Measurement:* % VO_2_ max and %1RM (Monitorisation: N/R) *HIIT:* *Interval:* 110 rpm at 100% VO_2_ max workload *Rest:* 75 rpm at 50% VO_2_ max workload *StrT:* 80% 1 RM Weight was increased when the participant was able to perform more than 10 reps on 2 consecutive sessions. *Pain:* N/R	2×/week 12 weeks	Maximal cardiopulmonary test on a bicycle. VO_2_ max, expiratory volume, respiratory exchange ratio, and HR were recorded. Parameters were adapted at 6 weeks with another cardiopulmonary test. 1RM testing was performed for every exercise.
	HIIT (AerT) + Core StrT	*HIIT protocol:* Same HIIT protocol as above. *Core muscle training:* 6 static core exercises [glute bridge, resistance band glute clam, lying diagonal back extension, adapted knee plank, adapted knee side plank, elastic band shoulder retraction with hip hinge): 2 × 10 rep of a 10 s static hold.	*Measurement:* % VO_2_ max and %MVC (Monitorisation: N/R) *HIIT:* *Interval:* 110 rpm at 100% VO_2_ max workload *Rest:* 75 rpm at 50% VO_2_ max workload *Core:* 40–60% of the MVC of m. transversus abdominis, m. multifidus, m. gluteus. Progressive increase of time and load. *Pain:* N/R		
	HIIT (AerT)+ Global and Core StrT	*HIIT protocol:* Same HIIT protocol as above. *High intensity StrT:* Same exercise as above: 1 × maximum 12 rep *Core muscle training:* Same exercise as above: 1 × 10 rep of a 10 s static hold.	*Measurement:* % VO_2_ max, %1RM and %MVC (Monitorisation: N/R) *HIIT:* *Interval:* 110 rpm at 100% VO_2_ max workload *Rest:* 75 rpm at 50% VO_2_ max workload *StrT:* 80% 1 RM Weight was increased when the participant was able to perform more than 10 reps on 2 consecutive sessions. *Core:* 40–60% of the MVC of m. transversus abdominis, m. multifidus, m. gluteus. Progressive increase in time and load *Pain:* N/R		
	HIIT (AerT)+ Mobility	*HIIT protocol:* Same HIIT protocol as above. *Mobility Training*: 6 mobility exercises (hamstrings stretch, gluteus medius stretch, lower back rotation mobilisation, back extension stretch, hip flexor stretch, and mid-back extension mobilisation): Stretches were held on each side 2 × 30 s, and mobilisations were performed 2 × 10 rep.	*HIIT:* *Interval:* 110 rpm at 100% VO_2_ max workload *Rest:* 75 rpm at 50% VO_2_ max workload *Mobility: * N/R *Pain:* N/R		

1RM, one-repetition maximum; ACSM, American College of Sports Medicine; AerT, aerobic training; BalanceT, balance training; bpm, beats per min; HIIT, high-intensity interval training; HR, heart rate; HR_max_, maximal heart rate; HRR, heart rate reserve; LA, lactate level; MICT, moderate-intensity continuous training; MVC, maximal voluntary contraction; N/A, not applicable; N/R, not reported; RPE, rating of perceived exertion; rpm, revolutions per minute; StrT, strength training; VO_2_ max, maximal oxygen uptake; WR_max_, highest work rate.

**Table 3:** Assessment of the studies’ quality based on the PEDro Scale.

Items												
	1	2	3	4	5	6	7	8	9	10	11	Total
Atan et al., 2020 [15]	1	1	1	1	0	0	1	1	1	1	1	8
Berg et al., 2020 [50]	1	1	1	0	0	0	0	1	1	1	1	6
Bressel et al., 2014 [44]	1	0	0	1	0	0	0	1	1	1	1	5
Flehr et al., 2019 [52]	1	1	1	1	0	0	1	1	1	1	1	8
Hanssen et al., 2018 [49]	1	1	1	1	0	0	1	1	1	1	1	8
Keogh et al., 2018 [45]	1	1	1	1	0	0	0	1	1	1	1	7
Sandstad et al., 2015 [51]	1	1	1	1	0	0	0	1	1	1	1	7
Sveas et al., 2014 [16]	1	1	1	1	0	0	1	1	1	1	1	8
Sveas et al., 2019 [49]	1	1	1	1	0	0	1	1	1	1	1	8
Thomsen et al., 2019 [53]	1	1	1	1	0	0	1	1	1	1	1	8
Verbrugghe et al., 2018 [47]	1	0	0	1	0	0	1	1	1	1	1	6
Verbrugghe et al., 2019 [17]	1	1	1	1	0	0	0	1	1	1	1	7
Verbrugghe et al., 2020 [48]	1	1	1	1	0	0	0	1	1	1	1	7

1, patient choice criteria are specified; 2, random assignment of patients to groups; 3, hidden assignment; 4, groups were similar at baseline; 5, all patients were blinded; 6, all therapists were blinded; 7, all evaluators were blinded; 8, measures of at least one of the key outcomes were obtained from more than 85% of baseline patients; 9, intention-to-treat analysis was performed; 10, results from statistical intergroup comparisons were reported for at least one key outcome; 11, the study provides point and variability measures for at least one key outcome.

**Table 4:** Summary of findings and quality of evidence (GRADE).

Certainty Assessment						No. of Participants		Effect		Certainty	Importance
Outcome (No. of Studies)	Study Design	Risk of Bias	Inconsistency	Indirectness	Imprecision	HIIT	Control	Relative (95% CI)	Absolute (95% CI)		
Pain intensity (7)	RCT	Not serious	Serious	Not serious	Serious	119	120	-	−0.73 (1.40–−0.06)	Low (+) (+)	Critical
VO_2 max_ (6)	RCT	Not serious	Not serious	Not serious	Serious	112	118	-	0.69 (0.42–0.97)	Moderate (+) (+) (+)	Critical
Disability (4)	RCT	Not serious	Not serious	Not serious	Serious	35	33	-	−0.34 (−0.92–0.24)	Moderate (+) (+) (+)	Critical
Quality of life (4)	RCT	Not serious	Serious	Not serious	Serious	53	44	-	0.40 (−0.80–1.60)	Low (+) (+)	Critical

CI, confidence interval; RCT, randomised controlled trial.

## Data Availability

Not applicable.
